# Comparison of Wearable and Clinical Devices for Acquisition of Peripheral Nervous System Signals

**DOI:** 10.3390/s20236778

**Published:** 2020-11-27

**Authors:** Andrea Bizzego, Giulio Gabrieli, Cesare Furlanello, Gianluca Esposito

**Affiliations:** 1 Department of Psychology and Cognitive Science, University of Trento, 38122 Trento, Italy; andrea.bizzego@unitn.it; 2 Psychology Program, School of Social Sciences, Nanyang Technological University, Singapore 639798, Singapore; GIULIO001@e.ntu.edu.sg; 3 HK3 Lab, Rovereto, 38068 Trento, Italy; cesare.furlanello@hk3lab.ai; 4 Lee Kong Chian School of Medicine, Nanyang Technological University, Singapore 639798, Singapore

**Keywords:** wearable devices, physiological data analysis, signal processing, multivariate analysis

## Abstract

A key access point to the functioning of the autonomic nervous system is the investigation of peripheral signals. Wearable devices (WDs) enable the acquisition and quantification of peripheral signals in a wide range of contexts, from personal uses to scientific research. WDs have lower costs and higher portability than medical-grade devices. However, the achievable data quality can be lower, and data are subject to artifacts due to body movements and data losses. It is therefore crucial to evaluate the reliability and validity of WDs before their use in research. In this study, we introduce a data analysis procedure for the assessment of WDs for multivariate physiological signals. The quality of cardiac and electrodermal activity signals is validated with a standard set of signal quality indicators. The pipeline is available as a collection of open source Python scripts based on the pyphysio package. We apply the indicators for the analysis of signal quality on data simultaneously recorded from a clinical-grade device and two WDs. The dataset provides signals of six different physiological measures collected from 18 subjects with WDs. This study indicates the need to validate the use of WDs in experimental settings for research and the importance of both technological and signal processing aspects to obtain reliable signals and reproducible results.

## 1. Introduction

The quantification of peripheral physiological nervous signals is a core step in measuring the functioning of the autonomic nervous system [1]. Being able to observe these phenomena in real-life and without constraints imposed by laboratory settings is a key reason for adopting WDs in scientific research [2]. Wearable technologies enable the acquisition and quantification of physiological signals in a wide range of contexts, from personal uses to industrial and scientific research [3,4,5]. In a general sense, wearable devices (WDs) are portable, non-invasive devices that allow the acquisition of physiological signals during daily life, without the need for external equipment [6]. The application of WDs is boosted by their improved portability (e.g., increasing miniaturization and battery life) [7,8], as well as by new materials [9,10] and layouts with reduced invasiveness; e.g., epidermal tattoos [11], wireless ring pulse oximeter [12], garments [13] and masks [14].

Besides their higher portability, WDs may have an apparent lower cost than clinical-grade devices, enabling their diffusion in commercial applications [15]. In contrast to their growing market availability, the adoption of WDs in research is still limited. Wearable technologies could represent novel tools in the study of human physiology and autonomic responses to the scientific community [1,16], paving the way for a new generation of experiments in which long-term monitoring and real-life ecological acquisitions are key aspects. In particular, a decrease in obtrusiveness while monitoring a subject’s behavior and daily activities is expected by adopting WDs instead of clinical devices [2]. Many scientific fields would benefit from the adoption of WDs for the long-term, unobtrusive recording of physiological signals. In particular, scientific use of WDs has been explored in affective computing [17,18], research on autism [3,19], interpersonal coupling [20,21] and psychology in general.

However, due to the technical constraints imposed by improvements in the miniaturization and autonomy of WDs, the data quality that can be achieved is typically lower and more subject to noise due to body movements [22,23,24]. Further, we need to consider the hurdles in extracting, preprocessing and analyzing the data from non-standard data interfaces: the high scientific “total cost of ownership”, as well as the possible requirement of manual preprocessing, which is also associated to a limited reproducibility of research.

It is therefore necessary to create a working protocol and resources to validate WDs as a reliable scientific tool before their use in research [25]. In particular, the validation should be based on open-access scientific software and include a comparison with a baseline of results from signals collected by medical-grade devices, within the same or very similar experimental settings. In practice, a battery of signal processing algorithms or their combination in a pipeline should be applied to signals collected by clinical-grade and wearable devices, with a lower level of precision accepted considering the type and purpose of application.

Similar works in the literature mainly target particular settings; for instance, the usage of WDs in schools [26] with a particular type of users [27]. Studies focusing on the comparison between clinical and WDs are usually application-specific; for instance, the study of physiological synchrony [28], sleep [29,30], vital signs monitoring [31] or usage in clinical settings [32,33].

However, despite its importance, the validation of a WD is often omitted or left to manufacturers, who rarely provide the dataset used and the details about the validation procedure. Although several datasets with physiological signals are available, such as DEAP [34], MANHOB-HCI [35], SEMAINE [36] and the PhysioBank archive [37], none of the existing resources provides signals collected from both wearable and clinical-grade devices to allow comparison.

Finally, the set of algorithms applied in the validation should be also available to allow reproducibility and for further reference.

In this study, we introduce scientific software and data aimed at validating the use of WD for research within different real-life contexts (resting and movement), focusing on cardiac and electrodermal activity signals. Our contributions are as follows: (a) the creation of the Wearable and Clinical Signals (WCS) dataset, which allows a representative set of WDs to be compared with clinical-grade devices; (b) a procedure to assess the validity of signals collected from WD; and (c) quantitative results on physiological signals of interest for the affective-computing field.

Physiological signals were collected from a clinical-grade device and from two WDs to allow both the direct comparison of the signals’ data quality and the investigation of the reproducibility. The adopted protocol comprises two different activities to evaluate the performance in different real-life contexts.

## 2. Methods

### 2.1. Participants

A total of 18 participants (with six males) were recruited for the experiment through announcements to university students (age: average = 27.6, SD = 7.04). The participation in the experiment was voluntary, and credits were granted for the fulfillment of an academic course. Before starting the experiment, each participant was briefed about the purpose and procedure of the experiment and gave informed consent. The experiment was approved by the Independent Review Board of the University of Trento (2017–2019) and was conducted according to the principles of the Declaration of Helsinki. All the data have been de-identified by the assignment of randomized numeric identifiers to each subject, and we removed any reference to the absolute timestamps.

### 2.2. Devices and Architecture

**Figure 1:**
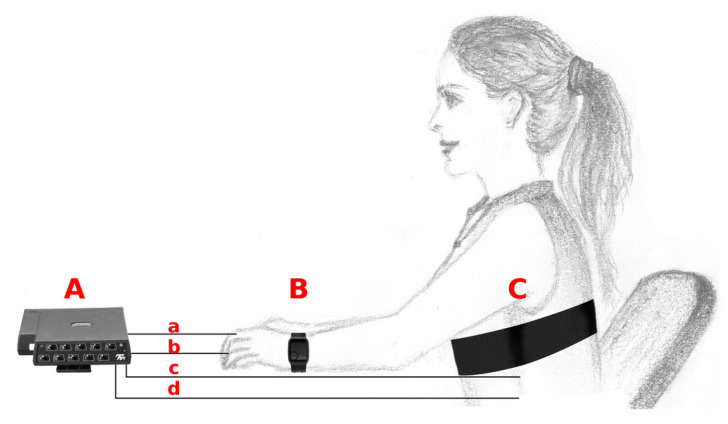
Illustration showing the devices used for the acquisition of physiological signals. **A**: Thought Technology FlexComp (picture from www.thoughttechnology.com) with four wired sensors: (**a**) electrodermal activity, (**b**) blood volume pulse, (**c**) electrocardiogram, (**d**) respiration; **B**: Empatica E4 (picture from www.empatica.com); **C**: ComfTech HeartBand.

The physiological signals were simultaneously collected by three devices (see Figure 1): the FlexComp unit, which provided the reference signals, and two WDs—the Empatica E4 and the ComfTech HeartBand.

The FlexComp acquisition unit was connected through an optical fiber to a signal converter, which in turn was connected to a Windows 10 workstation where the proprietary BioGraph Infinity Software Platform managed the acquisition and collection of the signals from the unit.

The two WDs sent the data through a Bluetooth connection to an Android (5.1) tablet (Samsung Tab A) where a specifically developed application gathered the signals and sent them to a database through a secure connection. The application interface also enabled the annotation of manual markers to allow the synchronization of the signals collected with the FlexComp and WDs.

Before starting the experiment, the signals acquired through the two WDs were visually inspected by activating the real-time plotting function.

The WDs used in the experiment were selected according to four requirements enable defined to their usage in everyday life:

1. Wearability: the device should be easily worn by the subject with no need to apply conductive gels, wired electrodes or similar preparations;
2. Streaming: the device should stream the data in real-time to an external collector through a Bluetooth connection;
3. Availability: the device should be commercially available at the moment of testing (prototypes, proof-of-concepts or custom devices were not considered);
4. Performance: the final choice should favor the device with a higher sampling rate and higher number of sensors to select the most advanced technological solution.

Wearability is the main characteristics of WDs: it facilitates the acquisition of signals, reducing the discomfort of users and the time required to set the experiment, and it allows the investigation to be expanded to everyday applications. In some experimental settings, WDs are expected to be set directly by the user, with no aids or supervision from an experimenter. For this reason, the WDs to be selected needed to be easily worn by the user, without requiring any special preparation of the device. Streaming capability is also a key feature of WDs: it allows synchronization and communication with other devices and allows real-time applications, which are fundamental in remote health monitoring. In the experimental settings of this study, a streaming cabability was also imposed by the specific sensing architecture, with one hub collecting the data from all wearable devices and synchronizing them with the data from the FlexComp unit. There are several types of WDs currently available, from prototypes to medical-grade devices. Prototypes devices can demonstrate a novel technological solution or are developed for a specific application. While some of these prototypes are made commercially available from start-ups or small companies, their usage requires some technological skills and, for this reason, they fail to comply with the “wearability” requirement. On the opposite side, medical-grade WDs are the most advanced solution, but were not considered in this study as these types of devices already undergo a set of validation studies to be certified as “medical-grade”. We focused instead on intermediate solutions that were commercially available and could be accessed even by researchers with limited technical skills. Finally, we selected those devices that, compared to similar solutions, offered the best performances in terms of signal acquisition. We considered the maximum sampling frequency and the number of different signals that could be collected by the same device.

After a preliminary phase in which a list of WDs was tested, we selected two devices that fulfilled all the requirements: the Empatica E4 and the Comftech HeartBand. The Empatica E4 was chosen as a representative of wristband devices, while the Comftech HeartBand represented the smart-garments category.

#### 2.2.1. Reference: Thought Technology FlexComp

The clinical device we used as a reference was the Thought Technology FlexComp unit (commercial code: T7555M). It is a customizable acquisition unit which provides up to 10 input slots that can be used to connect diverse physiological sensors. The maximal sampling frequency is 2048 Hz, which was adopted for the experiment, with 14 bits of resolution for each input. The physiological signals acquired with the FlexComp unit were as follows:

1. Electrocardiogram (ECG): using three electrodes placed over the left and right coracoid processes and below the ribs on the left, the signal is pre-amplified and filtered by the EKG sensor (T9306M) which returns a single channel read in millivolts. UniGel electrodes (T3425) are used as conductive means between the sensor and the skin;
2. Electrodermal activity (EDA): two finger bands with Ag–AgCl electrodes (SA2659) are placed on the second and fourth finger of the left hand and connected to the sensor (SA9309M). The skin conductance is measured in microSiemens (μS);
3. Blood volume pulse (BVP): the sensor (SA9308M) is placed on the third finger of the left hand. The relative amount of reflected infrared light is measured;
4. Respiration (RESP): a band is worn on the chest to measure the relative volumetric expansion by the elongation of an elastic patch (SA9311M);
5. Trigger (TRG): a handle with a button to generate electrical impulses used to manually mark the experimental events.

The signals collected from the FlexComp unit were the raw signals provided by the device, and no signal pre-processing was applied, except for the conversion to the signal units.

#### 2.2.2. Empatica E4

The Empatica E4 [38] is a multi-sensor wristband designed for real-life acquisitions of physiological signals. It has streaming (through Bluetooth Low Energy) and storage (internal flash memory) capability; communication with an Android smartphone is provided by the EmpaLink Software Development Kit, which is used by a customized application for signal acquisition. The collected signals are as follows:

1. BVP: four light-emitting diodes (LEDs) are used to generate light at two different wavelengths (green and red) and two photodiodes are used to measure reflected light. Using two wavelengths and an appropriate proprietary algorithm to preprocess the signals, the E4 aims at reducing motion effects and sensitivity to external sources of light. Sensors are placed on the bottom of the wristband case in firm contact to the skin, and the signal is sampled at 64 Hz;
2. EDA: two stainless steel electrodes are placed on the band to allow positioning on the inner side of the wrist. Skin conductance is measured in microSiemens at a 4 Hz sampling rate;
3. Acceleration (ACC): three axes of acceleration (range ±2 g) are measured at a 32 Hz sampling frequency;
4. Skin Temperature (ST): measured by an infrared thermopile placed on the back of the case, at a 4 Hz sampling frequency.

The collected signals were the raw signals provided by the device and no signal pre-processing was applied, except for the conversion to the signal units.

#### 2.2.3. ComfTech HeartBand

The Comftech HeartBand is an elastic band with embedded tissue electrodes and an acquisition unit that is connected to the band with two snaps. Streaming is based on Bluetooth 2.0, and the documentation to decode the hexadecimal messages from the device was provided by the manufacturer.

The collected signals are as follows:

1. ECG: using two tissue electrodes placed over the chest and connected to the acquisition unit, the signal is sampled (128 Hz), pre-amplified and filtered to return a single channel read;
2. ACC: three axes of acceleration are measured by a sensor embedded on the acquisition unit and sampled at 200 Hz.

The collected signals were the raw signals provided by the device and no signal pre-processing was applied, except for the conversion to the signal units.

### 2.3. Experimental Protocol and Procedure

After signed informed consent was given, the experimenter instructed the participant regarding the placement of WDs and the experimental settings. The setup of the FlexComp unit, the acquisition software and the positioning of the sensors was performed by the experimenter to ensure high-quality signals. Regarding the WDs, the participant was asked to place the devices themselves after having received detailed instructions from the experimenter. This procedure reflects the idea that WDs are to be used in real-life contexts, where the presence of an experimenter is not expected. In particular, the participant was allowed to adjust the positioning of both the Empatica E4 and of the ComfTech HeartBand to favor comfort during the experiment. However, the placement of the E4 wristband was inspected by the experimenter to ensure the device was not worn too tightly (which would have prevented physiological blood flow) or too loosely (which would have prevented the smartwatch case being in contact with the skin) to guarantee optimal conditions for correct BVP signal acquisition.

The experiment comprised two phases—baseline (300 s) and movement (300 s)—which were administered in the same order for all participants.

During baseline, the participants were asked to remain sitting still. The signals collected during this phase represented a reference for the maximal signal quality that could be achieved by WDs, as all external sources of noise that could affect the signal quality (such as moving artifacts, sensor displacement or detachment) were experimentally avoided. This condition is far from the real-life context in which the WDs are expected to be used, but it is necessary to isolate the effects of technical limitations and constraints from other causes of errors.

The effects of body movements could be better evaluated in the movement phase, which concluded the experiment. During this phase, the subject was asked to stand and simulate walking in place, as free movement was prevented by the wires connecting the medical-grade sensors with the FlexComp unit. The experimenter suggested moving naturally to replicate the intensity of usual walking. In particular, special attention was paid to the movements of the left hand as, due to the presence of sensors, participants tended to keep it still and near to the body. This experimental phase was expected to be more similar to real-life contexts, and it can be used to evaluate performances of algorithms for every-day applications, such as health monitoring, fitness and stress detection.

### 2.4. Preprocessing

The raw signals were preprocessed in order to appropriately format the data for the distribution and signal analysis. The first step was the synchronization of the two signal flows, from the FlexComp device and from the WDs. After the synchronization, the absolute timestamps of the samples were converted into the elapsed time since the start of the experiment (start of the baseline). Based on the TRG signal, the instants of the beginning and end of each experimental phase were extracted and used to split the signals. Then, each portion of the signal was separately saved. The acceleration signals, which were three-dimensional, were converted into uni-dimensional signals by the computation of the acceleration module.

Due to the sensitive nature of the data, the dataset has not been made public, but the collected signals are available to researchers that aim to create and test new algorithms for the automatic identification of noise and artifacts: https://doi.org/10.21979/N9/42BBFA.

### 2.5. Signal Quality Analysis

**Figure 2:**
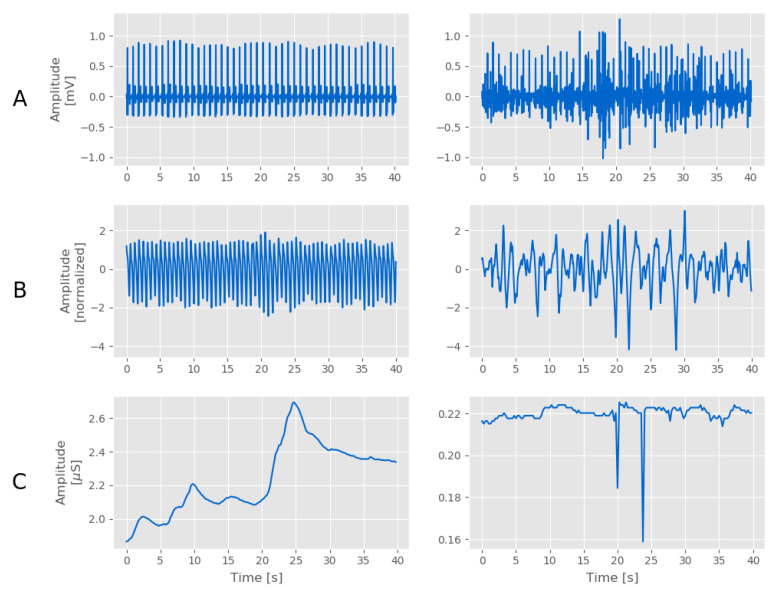
Portions of signals from wearable devices (WDs) extracted from the Wearable and Clinical Signals (WCS) dataset to show examples of high (left column) and low (right column) quality signals. (**A**): electrocardiogram (ECG) signals collected by the HeartBand; (**B**) blood volume pulse (BVP) signals collected by the E4; (**C**) electrodermal activity (EDA) signals collected by the E4.

Assessing the quality of the collected signals is crucial for reproducible research; this step is even more important when working with WDs, as signals are more sensitive to artifacts and noise due to body movements and technical constraints (see Figure 2).

**Figure 3:**
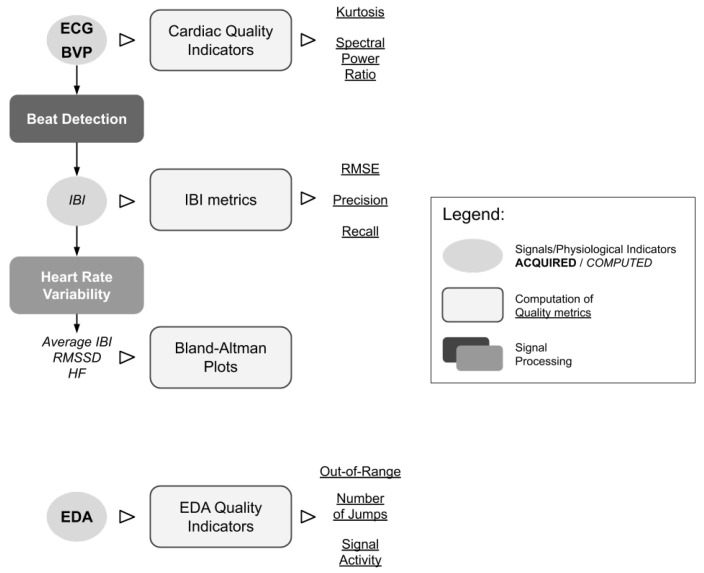
Data analysis procedure adopted in this study. IBI: inter-beat intervals; RMSSD: root of the mean of the squares of subsequent differences; HF: high frequency.

In this study, we adopted a data analysis pipeline (Figure 3) to investigate the quality of the acquired cardiac and EDA signals and of the derived signals and indicators. A set of signal quality indicators (SQIs) was defined to compare the quality of signals from clinical-grade and wearable devices.

The SQIs were computed for all cardiac signals and EDA signals of the baseline and movement sessions. A generalized linear model (GLM) was fit to investigate the contribution of the type of device (clinical or wearable) and session (baseline or movement) and their interaction on each SQI.

The processing pipeline was developed in Python, based on pyphysio [39], and it is publicly available at https://gitlab.com/abp-san-public/wearable-clinical-devices.

#### 2.5.1. Cardiac Signals

Several signal quality indicators (SQIs) have been proposed in the literature to evaluate the quality of cardiac signals. The SQIs can be grouped into two main categories: indicators that require a previous detection of heartbeats and indicators that are computed directly on the signal. We first used two SQIs from the second category to obtain an overall quantification of the signal quality and then adopted a specialized beat detection algorithm to assess the possibility of effectively identifying the heartbeats.

We used the kurtosis (*K*) and the spectral power ratio (ψ) which have been proposed for both the ECG [40,41] and the BVP [42,43]. Following the thresholds proposed in the literature, good quality signals are expected to have K>5 in the case of ECG [40] and K<3.5 in the case of BVP [44].

The spectral power ratio (SPR) ψ is defined as $\psi =\frac{PSD_{F_{1}}}{PSD_{F_{2}}}$, where $PSD_{F}$ is the spectral power in the frequency band *F*. $F_{1}$ is associated with the components in the signals that convey the information about the heart beats (the QRS complexes in the ECG and the pulses in the BVP); $F_{2}$ is the band which contains all the main frequency components of the signals. For ECG signals, $F_{1}=\left[5,14\right]$ Hz, $F_{2}=\left[5,50\right]$ Hz, [40]; for BVP signals, ($F_{1}=\left[1,2.25\right]$ Hz, $F_{2}=\left[0,8\right]$ Hz [42]). ψ is expected to have values 0.5≤ψ≤0.8 [40].

To compute the signal quality indicators (SQIs), the signal was resampled (128 Hz) and filtered (band pass filter: [0.5, 50] Hz). Then, the signal was standardized and segmented with non-overlapping windows of 5 s in length. The reported *K* and ψ represent the average value across all segments.

We then investigated the reliability of the acquired cardiac signals in terms of measuring the cardiac activity. To this end, we applied a beat detection algorithm to identify the heartbeats and derived the inter-beat interval (IBI) series—the series of distances between consecutive beats. The validation metrics were then computed by comparing the IBI series with the reference IBI series obtained from the ECG signal of the FlexComp, which were manually validated. These metrics are different from the SQIs presented before, as they are computed on a derived signal and not on the original cardiac signal, and thus the results depend also on the performance of the beat detection algorithm.

The ECG signals from the FlexComp unit were used to obtain the reference IBI series used for the validation: heartbeats were detected with the adaptive beat detection (ABD) algorithm (see Section 2.5.2) and then visually inspected to manually correct false detections and misdetections.

The ABD algorithm was also applied to the ECG signals of the Heart Band, while the derivative-based beat detection algorithm [45] was used to derive the IBI series from the BVP signals of both the FlexComp and E4. All IBI series were then processed to find detection errors by the application of the adaptive outlier detection (AOD) algorithm (see Section 2.5.3). In summary, for each subject, we obtained the reference IBI series ($IBI_{r}$) and those derived from the ECG signal of the Heart Band ($IBI_{HB}$), from the BVP signal of the FlexComp ($IBI_{FC}$) and from the BVP signal of the E4 ($IBI_{E4}$).

The quantification of the reliability of the beat detection was assessed in terms of the precision (*P*) and recall (*R*) of the detected beats and by the root mean square error ($e_{RMSE}$), by comparing each derived IBI series ($IBI_{d}$) with the $IBI_{r}$. To compute these metrics, we paired each beat $b_{d}$ of the $IBI_{d}$ to a beat $b_{r}$ of $IBI_{r}$. The pairing was considered valid if $|b_{d}-b_{r}|<0.5$ s. The beats that were successfully paired were considered true positives (TPs), the remaining unpaired beats in $IBI_{d}$ were considered false positives (FPs) and the remaining unpaired beats in $IBI_{r}$ were considered false negatives (FNs). Then, we counted the number of TPs ($n_{TP}$), the number of FPs ($n_{FP}$) and the number of FNs (($n_{FN}$)) to compute the overall precision $P=n_{TP}/\left(n_{TP}+n_{FP}\right)$ and recall $R=n_{TP}/\left(n_{TP}+n_{FN}\right)$. The RMSE was computed on paired beats: $e_{RMSE}=\sqrt{\frac{\sum_{TPs}{\left(IBI_{di}-IBI_{ri}\right)}^{2}}{n_{TP}}}$.

Finally, we evaluated the impact of the different types of WDs and cardiac signals on the main physiological indicators used to quantify the heart rate variability (HRV). For each IBI signal, we computed (a) the average of the IBI, (b) the root of the mean of the squares of subsequent differences (RMSSD) between the IBIs and (c) the relative power in the high-frequency (HF) band (between 0.15 and 0.4 Hz) [46]. The three HRV indicators were computed for each subject and session, on all cardiac signals. The HRV indicators computed from the IBI extracted from the ECG signals collected with the FlexComp unit were used as references. The HRV indicators computed from the IBI extracted from all other cardiac signals were qualitatively compared to the reference HRV indicators using Bland–Altman plots. Differences were reported in terms of percentages to allow the comparison of the performances of the different HRV indicators.

#### 2.5.2. Adaptive Beat Detection

The adaptive beat detection (ABD) algorithm applied to the ECG signal is composed of two steps:

i. Computation of the local range of the signal;
ii. Peak detection with threshold from the local range.

In the first step, the signal is segmented by windowing (window width: 1 s, overlap: 0.5 s) and the range is computed for each segment. The result is the estimation of the local range of the signal.

In the second step, the signal is scanned to find local maxima; a local maximum is considered a heart beat (i.e., R peak of the QRS complex) if it is followed by a local minimum and the difference is greater than 0.7 times the local range of the signal.

#### 2.5.3. Adaptive Outlier Detection

The AOD algorithm uses a fixed-size cache vector $IBI_{c}=\left(ibi_{1},ibi_{2},...,ibi_{k}\right)$ to store the last *k* valid IBI values in order to adapt to the variability of the IBIs. The size *k* is empirically set to 5 and the cache is initialized with the median value of the IBI series. Outlier detection is also regulated by the sensitivity parameter ϕ, which is empirically set to 0.25.

A detected beat is considered valid if its corresponding IBI value is within the interval [(1−ϕ)$ibi_{median}$, (1 + ϕ)$ibi_{median}$], where $ibi_{median}$ is the median of the values in $IBI_{c}$. When a new valid beat is detected, its IBI value is used to update $IBI_{c}$ using the first-in–first-out rule. $IBI_{c}$ is re-initialized when *k* consecutive non valid beats are detected.

#### 2.5.4. Electrodermal Activity Signals

Only a few studies have addressed the problem of assessing the quality of EDA signals as this is a quite recent issue associated with the emergence of wearable devices for EDA acquisition. The main approaches are based on the use of machine learning to classify high and low-quality portions of the EDA starting from a list of computed metrics [47,48], but without identifying the most appropriate metric that, alone, could be used as a SQI. On the other hand, these models are not very useful for signals from new devices as they need to be re-trained on new data that would require a manual labeling process.

*Quality metrics of EDA signals.* In this study, we adopted two metrics proposed by Kleckner and colleagues [25]: the ratio of out-of-range samples ($r_{oor}$) and the number of jumps, or drops, in the signal ($n_{j}$).

Following [25], we defined $r_{oor}$ as the ratio between the number of samples with values exceeding 60 μS or below 0.05 μS and the total number of samples. We empirically defined the threshold $r_{oor}=0.05$ to identify good ($r_{oor}\leq 0.05$) and bad ($r_{oor}>0.05$) signals; the selected threshold corresponded to a percentage of 5% of acquired samples that exceeded the physiological range for EDA signals.

A jump is defined in [25] as a portion of the signal in which the absolute value of the derivative of the signal is grater than 10 μS/s. This threshold was defined on signals sampled at 32 Hz, but the EDA signal from the Empatica was acquired at 4 Hz, which means that the 10 μS/s threshold was too high to allow the correct identification of the jumps. In addition, the amplitude of the EDA signal, and thus its derivative, also depends on the characteristics of the device, such as the amplification, type of electrodes and positioning. For these reasons, we derived a normalized version of the metric proposed by Kleckner and colleagues.

We first applied a low-pass filter to the EDA signal (cutoff frequency: 0.05 Hz) to remove the trend and the long-term components, obtaining the filtered signal ($s_{f}$), and then computed the normalized derivative $d_{n}=\frac{\left|{s_{f}}^{\prime }\right|}{\sigma_{s}}$, where $s_{f}^{\prime }$ is the derivative of the filtered signal and $\sigma_{s}$ is the standard deviation. Then, we considered the samples where $d_{n}$ is greater than 10 to be jumps. We considered a total number of five jumps over the 5 min sessions of the baseline and movement to be acceptable: therefore, signals with $n_{j}\leq 5$ (corresponding to maximum of one jump per minute of acquisition) were considered to be of good quality.

In addition, we defined a new metric, called the signal activity metric (α), to account for the cases in which the amplitude of the peaks associated with the galvanic skin response was too low or the peaks were not present (see Figure 2C). We defined α = $\sqrt{\frac{\sum_{n}s_{f}n^{2}}{N}}$, where $s_{f}$ is the filtered signal and *N* is the number of samples. As a threshold value, we selected α=0.05 μS, which is the minimal amplitude for a peak in the EDA signal to be considered as part of the galvanic skin response, as proposed in [49]. EDA signals with α<0.05 μS were considered to be of bad quality.

Before computing the SQIs, the EDA signals were filtered (low-pass filter, cutoff frequency $f_{lp}=1.5$ Hz). The EDA signals from the FlexComp device were first downsampled to 4 Hz to allow the comparison with the signals from the Empatica E4. The SQIs were computed on segments of the signals obtained from non-overlapping 20 s windows. The reported $r_{oor}$ and α values are the average of the values computed for each segment; $n_{j}$ is the sum of the jumps in each segment.

## 3. Results

### 3.1. Cardiac Signals

**Figure 4:**
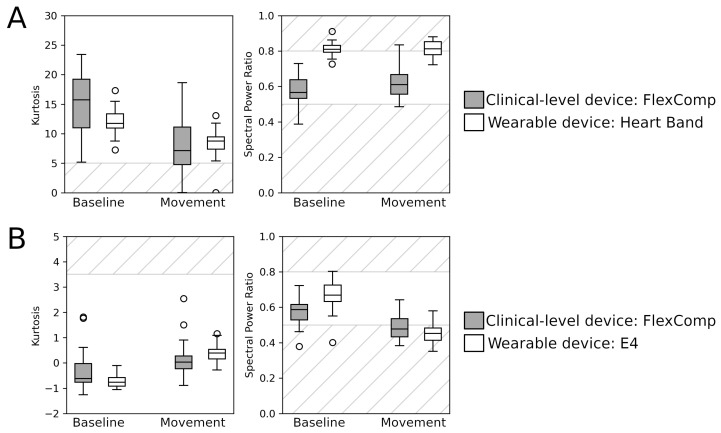
Signal quality indicators (SQIs) of the cardiac signals: (**A**). Kurtosis and spectral power ratio of the ECG collected from the FlexComp (gray) and Heart Band (white); (**B**). Kurtosis and spectral power ratio of the BVP collected from the FlexComp (gray) and E4 (white). Striped areas indicate the ranges of SQI values associated with low signal quality.

In general, the ECG signals collected with both the FlexComp and the Heart Band (Figure 4A) showed good quality in terms of kurtosis, although body movements caused a degradation; ECG signals from the HeartBand seemed to contain spurious frequency components, as indicated by the higher SPR.

The BVP signals from both the FlexComp and the E4 (Figure 4B) showed very good kurtosis; however, the low SPR for the movement session indicates that this signal is highly affected by body movements, as already reported in the literature [24].

For the ECG signals, kurtosis was found to be significantly affected by the type of session (p<0.001) and type of the device (p=0.035), but the latter result did not survive the Bonferroni correction for multiple tests. A significant contribution from the type of device was found on the SPR (p<0.001). For the BVP signals, a significant contribution of the type of device (p=0.038) and of the interaction between the type of device and session (p=0.020) was found on kurtosis; however, both results did not survive Bonferroni correction. On the SPR, a significant contribution was found for the type of device (p<0.001), type of session (p=0.001) and their interaction (p<0.001).

**Figure 5:**
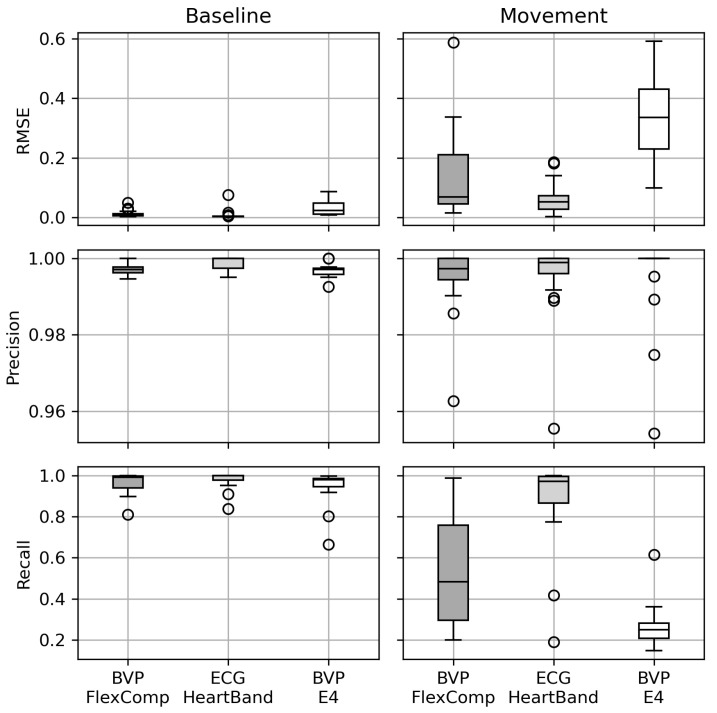
Metrics of the quality of the inter-beat intervals extracted from the cardiac signals.

During the baseline sessions, all devices showed good performances in terms of *P* and *R*, although the recall of the IBIs series derived from the BVP signals was inferior (see Figure 5. The $e_{RMSE}$ was also noticeably higher for the IBI series derived from the BVP signals. This result was expected, as the beat detection in BVP signals is in general more difficult due to posture and individual physiological characteristics [50]. As expected, all metrics worsened during the movement sessions; in particular, the IBI series derived from BVP signals of the Empatica E4 showed very low recall (below 0.4) and a high $e_{RMSE}$.

**Figure 6:**
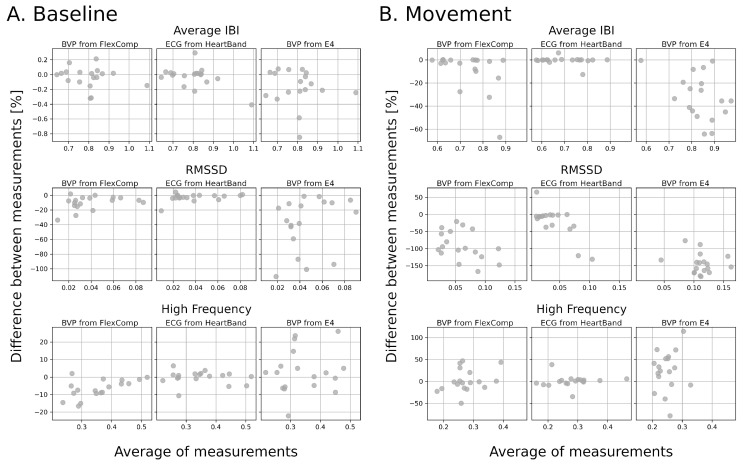
Bland–Altman plots to assess the reliability of the physiological indicators computed from the IBI signals extracted from different signals and devices. All plots refer to the indicators computed from the ECG signals collected by the FlexComp.

When considering the Bland–Altman plots of the physiological indicators extracted from the cardiac signals (Figure 6), it emerges that BVP signals show the greatest differences, with the E4 performing worse than the clinical-level device.

### 3.2. Electrodermal Activity

**Figure 7:**
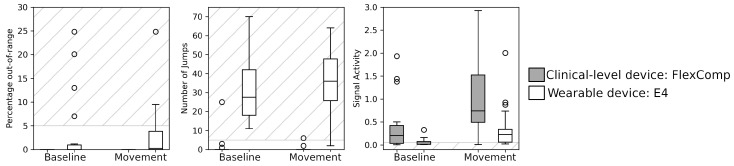
SQIs of the EDA signals collected from the FlexComp (gray) and E4 (white). Striped areas indicate the ranges of SQI values associated with low signal quality.

When we consider the SQI of the EDA signals (Figure 7), we observe that none of the EDA signals collected with the FlexComp presented out-of-range samples while few signals collected by the Empatica E4 appeared corrupted, with a worsening effect due to the body movements. In terms of the number of jumps, the FlexComp unit provided high-quality signals for both the baseline and the movement sessions, with few exceptions. In contrast, the signals collected with the Empatica E4 presented a low quality, with an average $n_{j}=31.6$ for the baseline and $n_{j}=36.7$ for the movement session: i.e., more than one jump every 10 s. The signal activity index of the signals collected with the Empatica E4 was low, despite the high number of jumps; in particular, for the baseline session.

The GLM revealed a significant contribution of the type of the device on the percentage of out-of-range samples (p=0.024, which did not survive Bonferroni correction), and on the number of jumps (p<0.001). Only the signal activity showed a main effect from the type of session (p<0.001).

## 4. Discussion

We adopted a set of SQIs derived from the literature to assess the overall quality of the cardiac signals and EDA signals provided in the WCS dataset.

In general, the signals collected with the clinical device showed an optimal signal quality in the baseline session, indicating that the experimental design and settings were appropriate for the acquisition of scientific-level data. During the movement session, the induced artifacts slightly worsened the signal quality, in particular for the BVP signal, which is known to be more sensitive to body movements. During the baseline, the wearable devices collected cardiac signals with a quality comparable to the signals from the FlexComp unit, but they were more affected by body movements, as shown by the worse signal quality in the movement session. For ECG signals, the kurtosis was more sensitive to a decrease in signal quality due to movements, while the SPR was capable of sensing differences between clinical devices and WDs. The SPR of BVP signals was influenced by both differences in the type of sessions (baseline and movement) and of devices (WDs and clinical); kurtosis was found to be less effective for quantifying differences between devices and sessions.

These patterns were confirmed when analyzing the precision of physiological indicators through Bland-Altman plots: the highest precision, during both baseline and movement precision, was achieved with the ECG signal from the Heart Band, while for the indicators computed from BVP signals, the precision was lower for both the FlexComp unit and E4. The signal quality of the EDA signals collected by the E4 did not appear to be appropriate for scientific research on our data: all SQIs detected significant differences between the quality of signals collected with the FlexComp unit and the E4, while only the signal activity indicator was shown to be sensitive to differences between the baseline and movement.

The lower quality of signals collected from WDs can be attributed to a number of technical limitations. For instance, the performance of some sensor components are reduced to decrease battery consumption and increase autonomy. In particular, the sampling frequency is kept lower than medical-grade devices, usually below 256 Hz, with consequent issues in the accuracy of the physiological indicators computed. To favor wearability, no aids are used to fix the sensors to the body (e.g., adhesive ECG electrodes). This allows for minimal shifts of the sensors that can introduce noise on the acquired signal [22,23,24]. Finally, although some anatomical loci are more appropriate than others for measuring a physiological signal, the need to embed the sensor in a wearable and comfortable support might constrain the positioning of the sensor into a non-optimal locus where the signal magnitude might be lower, such as the wrist instead of hand palm in the case of EDA [51].

This study evidenced the need to test and validate the use of WDs in experimental settings for research and the importance of both technological and signal processing aspects to obtain reliable signals. The validation should reflect the experimental context in which the data are collected; in particular, since WDs are employed to favor the freedom of movement of the subject, the influence of artifacts due to movements should be carefully considered.

The use of proper SQIs represents a quantifiable and reproducible method to identify signals with good quality. However, the evaluation of applicability should be done for each use case to account for the specific aim of the investigation and requirements in terms of the reliability of the computed metrics. The analysis of the Bland–Altman plots shows that different signals and devices are capable of different levels of precision of the computed physiological indicators. The scope of the analysis should be considered when assessing whether the precision required is compatible with the confidence ranges.

The collected data and the code to reproduce the pipeline are offered to the scientific community to encourage the development and validation of new SQIs and algorithms. The adoption of standardized metrics and procedures should favor the implementation of experiments with multivariate physiological signals, based on WDs.

As a final note, it is important to remark that the results of this study are not to be interpreted in terms of a technological validation of the WDs used for the experiment. The technological development is still in progress, and manufacturers of WDs are constantly improving sensing and acquisition technologies. Therefore, our results reflect the state of the technology at the time of the experiments. Nevertheless, the validation and quantification of the quality of the collected data is a critical step that should be performed each time the technology or the WDs are updated.
